# Short-chain fatty acids in cancer pathogenesis

**DOI:** 10.1007/s10555-023-10117-y

**Published:** 2023-07-11

**Authors:** Mark A. Feitelson, Alla Arzumanyan, Arvin Medhat, Ira Spector

**Affiliations:** 1 Department of Biology, College of Science and Technology, Temple University, Philadelphia, PA 19122 USA; 2 Department of Molecular Cell Biology, Islamic Azad University Tehran North Branch, Tehran, 1975933411 Iran; 3 SFA Therapeutics, Jenkintown, PA 19046 USA

**Keywords:** Short chain fatty acids, Cancer pathogenesis, Epigenetics, Immuno-regulation, Signal transduction

## Abstract

Cancer is a multi-step process that can be viewed as a cellular and immunological shift away from homeostasis in response to selected infectious agents, mutations, diet, and environmental carcinogens. Homeostasis, which contributes importantly to the definition of “health,” is maintained, in part by the production of short-chain fatty acids (SCFAs), which are metabolites of specific gut bacteria. Alteration in the composition of gut bacteria, or dysbiosis, is often a major risk factor for some two dozen tumor types. Dysbiosis is often characterized by diminished levels of SCFAs in the stool, and the presence of a “leaky gut,” permitting the penetration of microbes and microbial derived molecules (e.g., lipopolysaccharides) through the gut wall, thereby triggering chronic inflammation. SCFAs attenuate inflammation by inhibiting the activation of nuclear factor kappa B, by decreasing the expression of pro-inflammatory cytokines such as tumor necrosis factor alpha, by stimulating the expression of anti-inflammatory cytokines such as interleukin-10 and transforming growth factor beta, and by promoting the differentiation of naïve T cells into T regulatory cells, which down-regulate immune responses by immunomodulation. SCFA function epigenetically by inhibiting selected histone acetyltransferases that alter the expression of multiple genes and the activity of many signaling pathways (e.g., Wnt, Hedgehog, Hippo, and Notch) that contribute to the pathogenesis of cancer. SCFAs block cancer stem cell proliferation, thereby potentially delaying or inhibiting cancer development or relapse by targeting genes and pathways that are mutated in tumors (e.g., epidermal growth factor receptor, hepatocyte growth factor, and MET) and by promoting the expression of tumor suppressors (e.g., by up-regulating PTEN and p53). When administered properly, SCFAs have many advantages compared to probiotic bacteria and fecal transplants. In carcinogenesis, SCFAs are toxic against tumor cells but not to surrounding tissue due to differences in their metabolic fate. Multiple hallmarks of cancer are also targets of SCFAs. These data suggest that SCFAs may re-establish homeostasis without overt toxicity and either delay or prevent the development of various tumor types.

## Introduction

The human gut microbiome is an important interface between the body and the environment. It consists of a wide range of microorganisms that help to maintain homeostasis under normal conditions and in the face of stress, environmental pollutants, changes in diet, and exposure to toxins, antibiotics, and infectious agents [1]. Normal microbiota is important to properly metabolize food, provide essential nutrients (made by the resident microbiota for the host, e.g., vitamins), develop immunity against pathogens while suppressing immune responses against food antigens, and block the development of chronic inflammation which could increase risk for different cancers. It has recently been recognized that the gut microbiome impacts upon the pathogenesis of cancer by delaying or preventing cancer onset or cancer development [2]. In particular, short-chain fatty acids (SCFAs), which are metabolic products of selected gut bacteria, impact upon the appearance, and progression of many diseases, including cancers, by (i) attenuating inflammation and (ii) altering cellular gene expression by multiple mechanisms including epigenetic modification [3, 4].

**Fig. 1:**
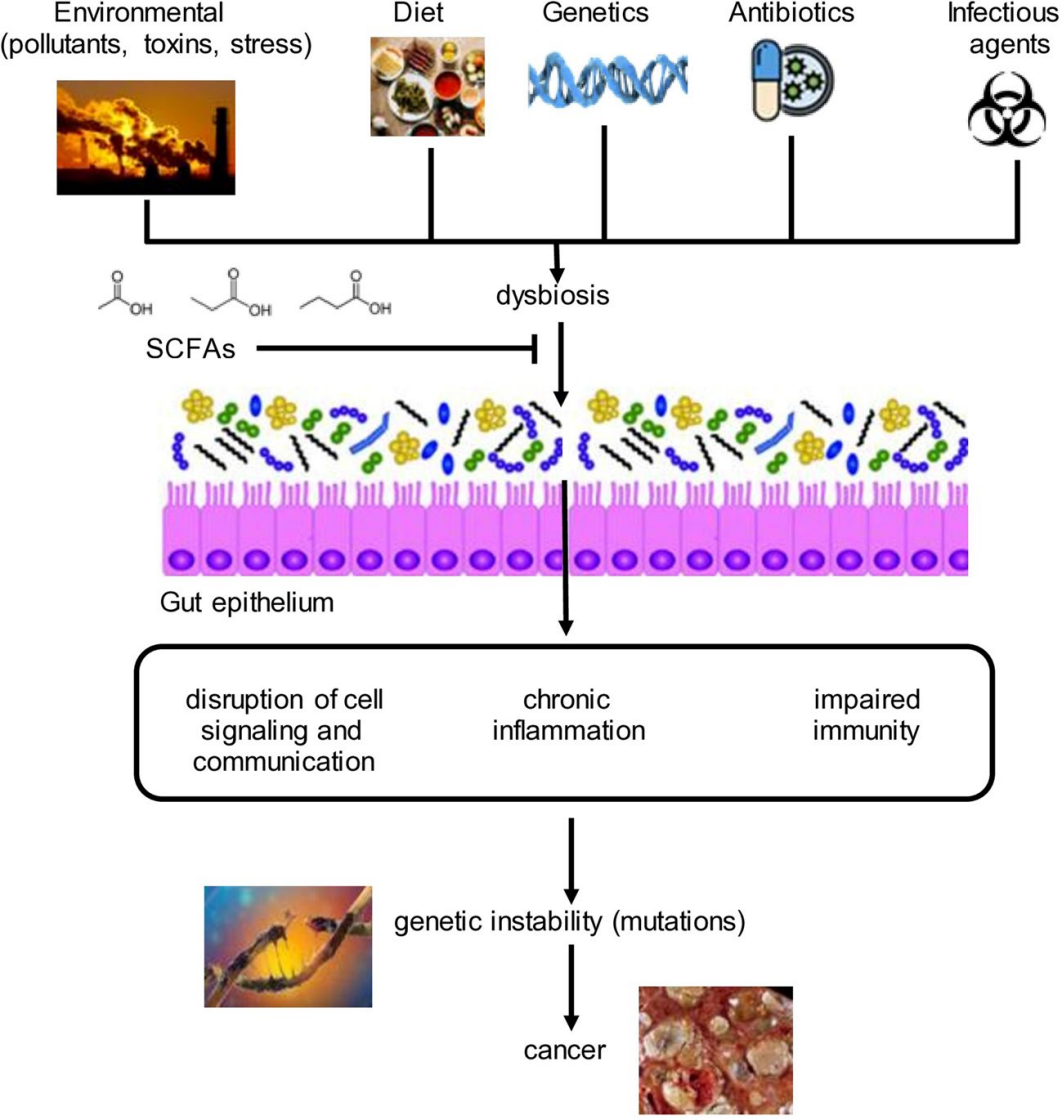
Normal colonocyte metabolism and energy production is restored by SCFAs. This is accompanied by the re-establishment of normal gut barrier function. The latter limits the penetration of pro-inflammatory bacterial molecules (e.g., lipopolysaccharides), toxins, pollutants, antibiotics and microbes through the gut wall. SCFA immunomodulation reduces the levels of reactive oxygen and nitrogen species. SCFAs also epigenetically alter gene expression in immunologically competent cells, thereby extinguishing the pro-inflammatory state. This down-modulates chronic inflammation and restores immunological homeostasis [15], thereby preventing disease progression and delaying the onset of cancer

SCFAs are simple aliphatic carboxylic acids 1–6 carbons in length. The most abundant SCFAs are acetate, propionate, and butyrate. They are made by selected gut bacteria that metabolize dietary fiber. SCFAs are absorbed via simple diffusion and by active transport of SCFA ions via transporters MCT-1 (or Slc16a1), [Na^+^]-coupled SMCT-1 (or SLC5A8), OAT2, and OAT7 [5]. SCFAs transporters and ligands are present in the membranes of virtually all cells/tissues, including immune cells [4, 5]. They are rapidly transported across the apical membrane of intestinal colonocytes. Some SCFAs (that are not consumed by the colonocytes for energy production) are transported across the basolateral membrane, enter the blood circulation, and may directly affect cells of numerous tissues [6]. SCFAs act as ligands for G-protein coupled receptors (GPCRs) [7, 8]. GPR43 (or FFAR2) has higher affinity for propionate, and GPR41 (or FFAR3) has higher affinity for butyrate. GPR109a (or HCA2) is activated only by butyrate [9]. SCFAs may enter and accumulate in the nucleus, where they act as (i) histone deacetylase inhibitors (HDACi) (where butyrate is the most potent HDACi among all known natural compounds). Mechanisms of HDACi include blocking the active site in HDAC and activation of GPCRs (which reduces expression of HDAC-encoding genes) [10–12] and (ii) modifying cell gene activity (where butyrate binds to butyrate-responsive elements in cellular gene promoters). This may explain the pleiotropic effects of butyrate [13, 14] (Fig. 1).

## Dysbiosis

Diet, as well as physical and psychological stresses, impact upon the composition of the bacteria in the gut, skin, nasal cavity, lungs, mouth, breast, stomach, colon, and urogenital tract [16]. This may result in dysbiosis, which is characterized by altered ratios of microbes, by diminished microbial diversity, and/or by the overgrowth of bacteria in parts of the gut and other tissues where they do not belong. Dysbiosis is often associated with chronic inflammation which is a major outcome of altered immunological homeostasis [16–19] (Fig. 1). For example, chronic inflammation in the colon is often accompanied by the presence of a “leaky gut” in which microbes and microbial products that are normally restricted to the gut lumen penetrate the intestinal epithelia and are then exposed to the underlying immune elements (in Peyer’s patches and lamina propria) as well as to the systemic immune system [20]. Overgrowth of *Escherichia coli*, *Bacteroides fragilis*, and/or *Fusobacterium nucleatum* in the colon produce toxins that mediate double stranded DNA breaks. While the latter is a defense mechanism destroying completing microbes, they also trigger the release of reactive oxygen and nitrogen species that contribute to host DNA damage via inflammation [16] (Fig. 1). *B. fragilis* also produces a toxin that cleaves the cell–cell adhesion molecule, E-cadherin. This results in the release of the E-cadherin binding protein, β-catenin, which accumulates in the cytoplasm and nucleus, resulting in altered patterns of host gene expression that promote tumorigenesis [16]. Normally, intact epithelial surfaces separate non-toxin-producing microbes from the immune system, but when these epithelia are compromised, invading microbes are detected by various pattern recognition receptors that trigger the production of pro-inflammatory cytokines that mediate tissue damage. In another example, *Lactobacilli* are a hallmark of a healthy female reproductive tract by preventing invasion of pathogenic microbes such as *Atopobium vaginae* and *Porphyromonas* sp., which are associated with endometrial cancer [16]. Other tumors arising on the background of chronic inflammation include cancers of the rectum, breast, lung, head and neck, liver, pancreas, bladder, esophagus, and ovaries [16–19, 21, 22]. Thus, dysbiosis among tumor-bearing patients is often characterized by diminished levels of gut bacteria making SCFAs [23, 24]. SCFAs have both anti-inflammatory and anti-tumor properties [25, 26]. Thus, inhibition of inflammation by SCFA-mediated immunomodulation is expected to reduce the risk of cancer development (Fig. 1). Since tumor cells display genetic instability, therapeutic targeting of cancer cells will ultimately select for resistance and relapse, but immunomodulation, especially prior to tumor appearance, may be a viable approach to attenuating the pathogenesis of cancer. If so, then restoration of physiological levels of SCFAs may block tumor development and/or progression by ameliorating inflammation and dysbiosis [27] (Fig. 1).

SCFAs contribute to homeostasis, in part, by promoting the production of mucins and of anti-microbial peptides (e.g., α-defensins) in the gastrointestinal tract [5, 28], both of which restrict microbes to the gut lumen, thereby preventing bacterial penetration through the gut epithelium which would otherwise potentially trigger chronic inflammation. Mucins consist of a family of proteins, some of which promote carcinogenesis. However, some are transcriptionally up-regulated by β-catenin, which may contribute to homeostasis [29]. α-Defensin levels are often depressed in dysbiosis and associated chronic inflammation [30] but are restored by butyrate [31]. Thus, the restoration of microbial defenses and gut integrity (i.e., healing of the “leaky gut”) by SCFAs alleviates dysbiosis and chronic inflammation by promoting immunological homeostasis.

Dysbiosis is also often found associated with epithelial to mesenchymal transition (EMT) and metastasis [16, 22]. A defining characteristic of EMT is resistance to anoikis, which is a form of programmed cell death that occurs in anchorage-dependent cells when they detach from the surrounding extracellular matrix (ECM). EMT promoting pathogens block cell–cell junction proteins by producing proteases that cleave E-cadherin and other proteins that contribute to cell adhesion, causing disruption of cell polarity and loss of tissue morphology. In gastric cancer, *Helicobacter pylori* overgrowth compromises gastric epithelia, induces “stemness” via activation of β-catenin [32] and facilitates both morphological transition to a mesenchymal phenotype and migration, which are characteristic of EMT [33]. Severe inflammation precedes EMT by overwhelming both dendritic cells and T regulatory (Treg) cells that immunomodulate inflammation, as documented in the colon, urogenital tract, and other anatomical sites [34]. In this context, pelvic inflammation was commonly observed among prostate cancer patients with aggressive disease, and this was also associated with markers of EMT [35]. Thus, chronic inflammation and dybiosis are risk factors not only for cancer initiation but also correlates with EMT and cancer progression.

## Chronic inflammation and “prevention by delay”

Inflammation could be triggered by many different means, including bacterial and viral infections, autoimmune diseases, obesity, tobacco smoking, asbestos exposure, and excessive alcohol consumption. The accumulation of cancer predisposing mutations in oncogenes, tumor suppressor genes, DNA repair genes, and genes responsible for regulating homeostasis via epigenetic mechanisms also contribute to inflammation [21]. Since many tumor types develop on a background of chronic inflammation, immunomodulation of chronic inflammatory diseases may alter their pathogenesis so that tumor appearance is either delayed or prevented. In this context, the concept of “prevention by delay” encompasses therapeutic intervention prior to tumor appearance (Fig. 1), with the aim of helping people live out their lifespan cancer free [36]. This concept is premised on the idea that milder treatments (e.g., with SCFA-based formulations) over a long period of time may ameliorate chronic inflammatory diseases and be more effective in reducing morbidity and mortality than the cytotoxic therapies used for treating cancer today [36]. The fact that SCFAs are normally made in the gut of healthy individuals and are generally regarded as safe suggest that their therapeutic benefits will not be accompanied by side effects, thereby providing strong rationale for their use in patients suffering from chronic inflammatory diseases.

In chronic hepatitis B, for example, integration of hepatitis B virus (HBV) sequences into regenerating infected hepatocytes following repeated bouts of hepatitis results in elevated expression of the virus oncoprotein, hepatitis B x (HBx), which constitutively activates many pro-inflammatory pathways such as nuclear factor kappa B (NF-κB) [37, 38], thereby promoting chronic liver disease (CLD). Thus, the strategy here would be to ameliorate the pathogenesis of CLD by immunomodulation to limit the progression of CLD (by reducing inflammation, apoptosis, and regeneration) which reduces the risk for hepatocellular carcinoma (HCC) development. In fact, this has been recently demonstrated [39].

In CLD, HBx expression and activity are stimulated in an environment where active immune responses trigger persistent oxidative stress [40]. HBx also promotes oxidative stress through the expression of pro-inflammatory cytokines [41] and is inhibited when oxidative stress is reduced [42]. Further, stimulation of GPR43, which binds to multiple SCFAs (acetate, propionate, and butyrate), blocks the ability of HBx to stimulate NF-κB [43], thereby attenuating these pro-inflammatory and pro-carcinogenic signaling pathways [43, 44]. Recently, SCFA feeding significantly reduced the incidence of liver cell dysplasia and HCC in HBx transgenic mice [39], suggesting that immunomodulation of CLD by SCFAs may reduce the risk of developing cancer. This approach may also be valuable in altering the pathogenesis of other cancers. For example, supplementation with SCFAs for kidney and liver diseases, inflammatory bowel disease, and colon cancer reduced the pro-inflammatory mediators, tumor necrosis factor alpha (TNFα), interleukin-6 (IL-6), and C-reactive protein, as well as disease progression [45]. More than 20% of tumor types arise on a background of chronic inflammation [46] so that “prevention by delay” may effectively reduce cancer risk [36].

## Why SCFAs instead of probiotic bacteria?

The development of SCFAs as immunomodulatory therapeutics would provide numerous advantages over other microbiome-based approaches, even though modulation of the gut microbiome by prebiotics (i.e., fiber-rich foods that support healthy gut bacteria), probiotics (i.e., selected bacteria), or fecal transplants are areas that are being actively pursued for various diseases characterized by dysbiosis [17]. However, there are variables precluding the success of probiotics, including differences in bacterial colonization efficiency in the gut and uncertainty about the persistence and production of the appropriate metabolites by the probiotic bacteria at high enough levels for a long enough period to have a sustained therapeutic effect. For these reasons, it will be difficult to develop reproducible probiotic-based therapeutics [47]. In addition, bacteria from other anatomical locations (skin, oral cavity, urogenital tract, etc.) may have an impact on cancer risk [48–51]. It is not clear whether probiotics introduced into the gut will have any impact on resident bacteria in these other locations. Since selected SCFAs have anti-inflammatory properties [4] and maintain homeostasis by immunomodulation [27], their development as therapeutic agents will overcome the limitations of probiotic bacteria outlined above and either delay or prevent the progression of chronic inflammatory diseases to cancers in the corresponding target tissues. Thus, the pleiotropic properties of SCFAs may be exploited to evaluate a “prevention by delay” approach to cancer pathogenesis by reducing cancer risk.

## SCFA-targeted pathways in carcinogenesis

Cancer is a multi-step process, and one of the advantages of using SCFAs is that they impact the expression of multiple genes and pathways, some of which are relevant to cancer. This is in contrast to the use of current anti-tumor compounds that target a single molecule or pathway. Multi-step carcinogenesis involves both driver mutations [52] and epigenetic changes in gene expression [53]. Driver genes impact the activity of multiple signaling pathways that regulate cell fate, cell survival, and genome maintenance [54, 55]. Genetic alterations in genes that determine cell fate (such as Wnt, Hedgehog, and Notch) [55] alter the balance between cellular differentiation and proliferation, favoring sustained proliferation, which is a hallmark of cancer. Global changes in the epigenetic landscape, which include inactivation of tumor suppressors and activation of oncogenes, are also hallmarks of cancer [56]. Since genetic and epigenetic changed in gene expression are mitotically heritable, they contribute importantly to tumor pathogenesis. SCFAs mitigate many of the epigenetic changes that contribute to cancer, suggesting that SCFA intervention in patients at high risk for tumor development may delay or prevent tumorigenesis at the molecular and cellular levels prior to the appearance of one or more cancer driver mutations.

### SCFAs and Wnt signaling

**Fig. 2:**
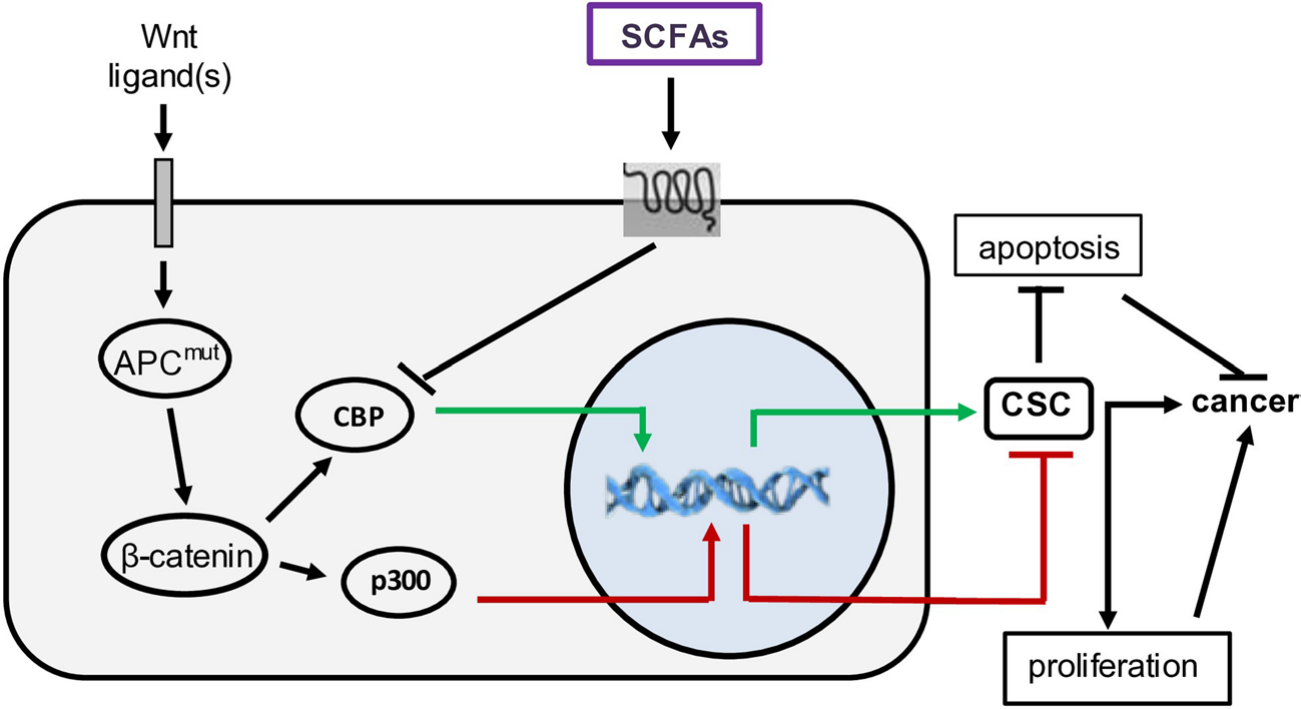
Application of SCFAs overcomes the constitutive activation of Wnt signaling by mutational inactivation of APC (APC^mut^), which normally degrades β-catenin. β-Catenin activates the transcription factor CBP, which alters gene expression that promotes the development of cancer stem cells (CSC) (in green) leading to malignancy. CBP is inhibited by SCFAs which attenuate the development of CSCs and malignancy. β-Catenin also promotes the activity of the transcription factor, p300, which alters patterns of gene expression in the nucleus that blocks the development of CSCs (in red). Thus, the role of SCFAs is to change the impact of Wnt signaling from pro-carcinogenic to anti-carcinogenic

The adenomatous polyposis coli (APC) protein is part of the Wnt pathway, which normally degrades β-catenin, but when APC is mutated, β-catenin accumulates in the cytoplasm and nucleus, where it may contribute to the pro-tumorigenic phenotype characterized by “stemness” and resistance to apoptosis. One study showed that butyrate did not directly impact the expression of Wnt target genes, but up-regulates the expression of retinoic acid, which promotes cell differentiation of intestinal epithelial cells [57]. The latter is partially mediated through Wnt [58], suggesting that SCFA regulation of Wnt may promote differentiation in the place of “stemness” [59]. Independent observations have shown that butyrate increases cancer cell differentiation through Wnt signaling [60] (Fig. 2). If SCFAs help to maintain gut integrity by up-regulating β-catenin signaling in intestinal stem cells [61], then the modulation of β-catenin activity by SCFAs may re-establish intestinal homeostasis. The impact of SCFA up-regulated expression of β-catenin is modulated by the presence of its binding partners in the nucleus. β-Catenin-p300 complexes promote cell differentiation or apoptosis, while β-catenin-CBP (cAMP response element binding protein) complexes promote cell proliferation [60] (Fig. 2). These complexes epigenetically alter gene expression, since both p300 and CBP are acetyltransferases that target different gene networks [62]. In the liver, CBP maintains homeostasis in response to changes in nutrient levels by epigenetically regulating lipogenesis and gluconeogenesis [63], both of which are altered in cancer. Modulation of gene expression in the Wnt and other signaling pathways (Fig. 2) is mediated by HDAC inhibition [11, 60, 64]. This suggests that SCFAs alter cancer pathogenesis before tumors arise by impacting upon cell fate by promoting Wnt-mediated differentiation and inhibiting proliferation.

SCFAs also increase the methylation of oncogenes, thereby reducing their expression [60]. In glioblastoma, for example, methylation of the oncogene, HEY1, was associated with increased DNA methyltransferase (DNMT) and decreased HDACi activities. In addition, SCFAs alter acetylation status and activity of non-histone proteins such as NF-κB, MyoD, p53, and nuclear factor of activated T cells (NFAT) [10, 65]. Downstream consequences of these epigenetic alterations blocked Notch signaling, phosphoinositide 3-kinase (PI3K), and pro-oncogenic PI3K targets, B cell lymphoma 2 (bcl-2), hypoxia-inducible factor-1 alpha (HIF-1α) [66], and mammalian target of rapamycin (mTOR) [60, 67]. SCFAs block HIF-1α stimulation of tumor survival and growth under hypoxic conditions, but in immune cells, SCFA stimulation of mTOR signaling regulates T cell fate [68]. These and other epigenetic alterations in gene expression underscore their importance in the pathogenesis of cancer and also point to the potential roles of SCFAs in the delayed onset and possible treatment of already established tumors. Many of the genes that accumulate driver mutations encode proteins that regulate epigenetic changes in global gene expression [69]. This finding further highlights the importance of epigenetic regulation in maintaining cellular homeostasis, which is lost in malignant transformation.

### SCFAs and Hedgehog (Hh) signaling

Hedgehog (Hh) signaling is central for the determination of cell fate (e.g., differentiation) during embryogenesis, and in cancer, cell fate is being altered. During embryogenesis, this process is temporarily and spatially organized, but in cancer, the reactivation of this pathway is not organized, in part, due to the presence of driver mutations, resulting in persistent and disorganized growth. Valproate, a SCFA, inhibited Hh signaling and the proliferation of multiple myeloma cells [70]. However, butyrate promoted the differentiation of gastric cancer cells by increasing Hh and attenuating Wnt signaling [71] through changes in DNA methylation and histone acetylation [72]. These differences may be due to a change of balance between activator and repressor forms of glioma-associated oncogene (Gli) transcription factors in the Hh signaling pathway [73]. In gastric cancer, butyrate up-regulated the expression of secreted frizzled related protein in the Hh pathway, which is a natural inhibitor of Wnt signaling. Butyrate mediated demethylation and histone acetylation at the promoter region of SFRP, thereby restoring SFRP expression [72]. The latter suppressed the activity of T-cell factor/lymphoid enhancer factor (TCF/LEF), resulting in suppressed transcription and expression of β-catenin and Wnt target genes [72]. Butyrate also regulated other post-translational modifications, such as histone phosphorylation [74] and hyper-acetylation of non-histone proteins [10, 65, 75]. These results imply that the anti-cancer effect of SCFAs regulate cell fate determining pathways and that the outcome of a signaling pathway also depends on pathway crosstalk [76].

### SCFAs, fibroblast growth factor receptor 2 (FGFR2), and Hippo signaling

**Fig. 3:**
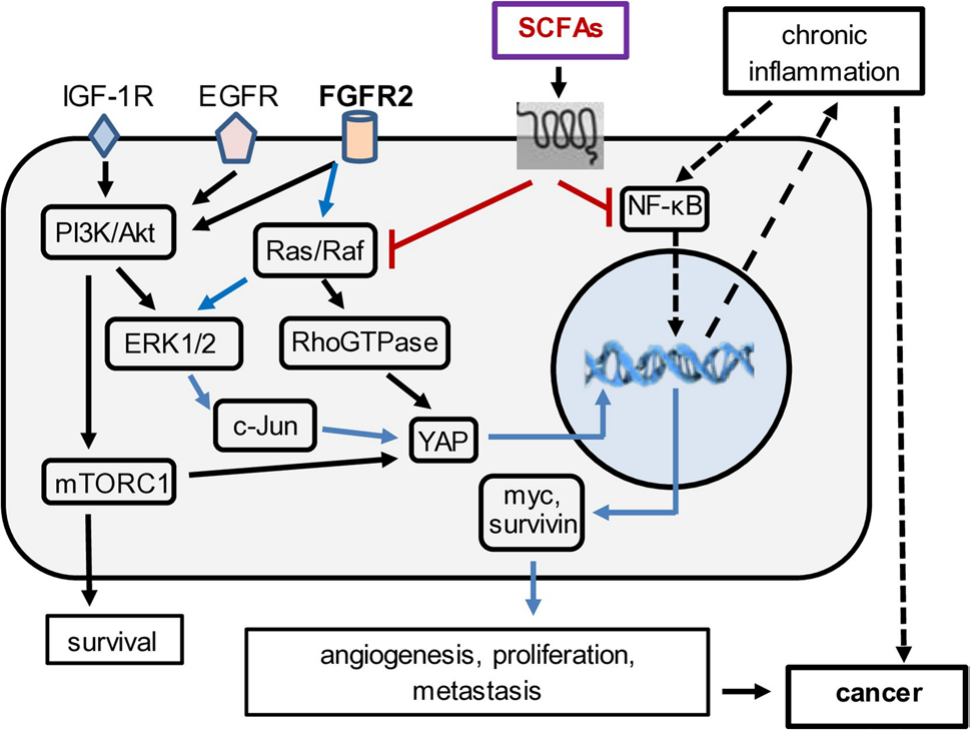
Putative impact of SCFAs upon FGFR2 and Hippo signaling. FGFR2 stimulates several pro-oncogenic pathways including PI3K/Akt and Ras/Raf. Constitutive activation of these pathways activates YAP, a component of the Hippo signaling pathway. YAP transcriptionally activates a number of pro-oncogenic genes, including myc and survivin, which promote the development of cancer (blue arrows). Chronic inflammation and accompanying intracellular oxidative stress stimulate NF-κB activity, which then enters the nucleus and transcriptionally activates many pro-inflammatory genes that exacerbate chronic inflammation. The latter promotes the generation and persistence of free radicals which are mutagenic and contribute to cancer development (dashed arrows). SCFAs block Ras/Raf signaling, not only by FGFR2 but also by other receptors such as EGFR and IGF-1R that signal through the same pathways. In this case, YAP activation is attenuated, thereby reducing the risk of carcinogenesis. SCFAs also strongly inhibit NF-κB, thereby extinguishing chronic inflammation and reducing the risk of progression to malignancy

FGFR2 is a receptor tyrosine kinase that induces proliferation, survival, and migration. Point mutations often result in the constitutive activation of FGFR2 signaling [77] in a variety of cancers (e.g., of the breast, lung, stomach, uterus, and ovaries) [78]. In gastric cancer, FGFR2 signals through the PI3K and Ras/Raf pathways [78]. Activation of Ras results in downstream constitutive activation of the transcription factor c-Jun [79], which transcriptionally activates Yes-associated protein (YAP) [80] (Fig. 3), which is part of the Hippo signaling pathway. YAP transcriptionally activates c-myc and other genes involved in carcinogenesis [81]. SCFAs block Ras signaling in HBx transgenic mice which delays the appearance of HCC [39] and triggers apoptosis in Ras transformed rat liver epithelial cells [79]. In the pathogenesis of colon cancer, butyrate down-regulates c-myc activity by decreasing c-myc-induced miR-17-92a cluster transcription [60, 82], which reduces colon cancer cell proliferation. SCFAs also up-regulate the c-myc cyclin-dependent kinase inhibitor (CDKi), p57, triggering cell cycle arrest [60]. Independent observations have shown that expression of the CDKi, p21^WAF1^, is up-regulated by butyrate [67]. Since p21^WAF1^ is a downstream target of wild type but not mutant p53, SCFAs may be able to inhibit proliferation and stimulate differentiation in cells that develop driver mutations and promote carcinogenesis. In addition, the finding that SCFAs attenuate Ras signaling *in vivo*, in part, by up-regulating the expression of human disabled 2 (DAB2), a tumor suppressor of the Ras and Wnt pathways [39], suggests that SCFAs may also impact FGFR2 and Hippo signaling in tumor development. Independent observations showed that butyrate induced growth arrest and apoptosis in mutant Ras activated cells by inhibition of extracellular signal–regulated kinases 1/2 (ERK1/2) and Akt phosphorylation [79], suggesting multiple inhibitory mechanisms are likely to be operative. Again, the pleiotropic properties of SCFAs, which simultaneously target multiple pathways that promote tumor development, provide strong rationale for their use prior to the appearance of cancer.

The G-protein coupled receptor, GPR43, binds butyrate, propionate, and acetate. Downstream signaling inhibits NF-κB activity, thereby reducing inflammation and the risk for tumorigenesis [83] (Fig. 3). In addition, GPR43 engagement by acetate augments Rho GTPase signaling, resulting in the stabilization and nuclear localization of YAP/TAZ transcriptional co-activators in the Hippo signaling pathway [83]. Importantly, in pancreatic cancer, the YAP/TAZ pathway is downstream from KRAS, PI3K, mTORC1/2, and the epidermal growth factor receptor (EGFR) [84]. PI3K and downstream signaling have been shown to be inhibited by SCFAs in colon cancer [64], in a sorafenib-resistant liver cancer cell line [85], in cervical cancer cells [86], and in Burkitt’s lymphoma cells [87]. G-protein coupled receptor agonists (such as acetate) signal to YAP/TAZ through ERK1/2 while insulin and insulin-like growth factor 1 (IGF-1) signal to YAP/TAZ through PI3K/Akt [84]. Both ERK1/2 and PI3K/Akt are modulated by SCFAs. Thus, acetate also modulates Hippo signaling in carcinogenesis.

### SCFAs, Notch signaling, and “stemness”

Like Wnt and Hh, Notch signaling is altered in a variety of cancers, and depending upon circumstances, may act as a tumor promoter or tumor suppressor [67, 88]. There are four Notch receptors that contribute to cancer. Notch 1 and 3 promote cell proliferation and metastasis; Notch 2 is constitutively activated in tumors; and Notch 4 is active in EMT, which also contributes to metastasis. Notch activation is important for promoting cancer in the liver, breast, and colon [89–91], among others. In contrast, Notch signaling acts as a tumor suppressor in thyroid cancer, skin cancer, and neuroblastoma [67]. Valproic acid, a SCFA, has been shown to inhibit the growth of ovarian, breast, liver, pancreatic, non-small-cell lung, and prostate cancers via modulation of Notch signaling [67]. Butyrate triggered growth arrest and cell differentiation and inhibited DNA synthesis in colon, prostate, and breast cancer cell lines [67]. In liver cancer, valproate inhibited tumor growth by down-regulating Notch signaling [92], while valproic acid inhibits cervical cancer by stimulating Notch signaling [93]. Therefore, this approach appears to alter cell fate mediated by Notch activation in multiple tumor types where Notch activation promotes tumorigenesis.

In hepatitis B infection, HBx has been shown to promote the development of “stemness” [94, 95], which is a central characteristic of CSCs. Specifically, HBx up-regulates the expression of Oct-4, Nanog, Klf-4, β-catenin, and the epithelial cell adhesion molecule (EpCAM) *in vitro* and *in vivo* [94]. HBx also stimulates Wnt, Notch, and Hh signaling in hepatocarcinogenesis [96–99]. Signal crosstalk among Wnt, Notch, and Hh contributes to the pathogenesis of many tumor types [100]. In addition, since these pathways participate in conferring “stemness” via self-renewal of CSCs [76, 101], their altered signaling may explain part of the mechanism whereby hepatitis B contributes to liver cancer. Given that SCFAs inhibit stem cell proliferation [102], and by extension, the proliferation of CSCs, this may contribute to their strong anti-tumor properties. Independent observations have shown that hematopoietic stem cells (HSC), through the accumulation of successive mutations, develop into CSCs that expand into acute myeloid leukemia (AML) [103] and that this expansion can be inhibited by valproic acid and other HDAC inhibitors [104]. Since CSCs are responsible for relapse in a variety of tumor types (including AML), SCFAs may find clinical application in delaying or preventing tumor relapse. However, crosstalk between these various cell fate determining pathways (Wnt, Hh, and Notch) [76] means that SCFAs may block or promote the development of tumors depending upon their binding partners in the cell (e.g., p300 or CBP), cell type, dosage, and duration of treatment. Thus, their efficacy needs to be carefully evaluated in human clinical trials.

## SCFA impact upon the microbiome

SCFAs are not only produced by various gut microbes but also impact upon the composition of gut bacteria and gut barrier integrity, either alone or in combination with other ingested compounds. For example, butyrate activates the aryl hydrocarbon receptor (AhR) [105], which is a transcription factor that promotes xenobiotic metabolism. The latter activates cytochrome p450 members that metabolize aryl hydrocarbons, thereby promoting homeostasis. Oral administration of graphene oxide potentiates the effects of butyrate on cytochrome p450 activation via AhR signaling [106]. Activated AhR inhibits inflammation by down-regulating the pro-inflammatory Th17 response [107] and contributing to the maintenance of intraepithelial lymphocytes (IEL), thereby contributing to a stable gut microbiome [108]. AhR activation also facilitates the development of Tregs while disruption of AhR signaling results in altered gut microbial composition [106]. Thus, dietary exposure to graphene oxide in combination with butyrate has an impact upon the composition of the gut microbiome. Diet is also a major contributor to the composition of the gut microbiome in that a high-fat diet is associated with low-grade chronic inflammatory conditions such as obesity, diabetes, and non-alcoholic steatohepatitis, all of which are associated with intestinal dysbiosis. These and many other chronic inflammatory diseases are often characterized by decreased production of SCFAs, suggesting that SCFA administration may re-establish immunological homeostasis by attenuating chronic inflammation.

## SCFAs epigenetically target many genes/pathways that are mutated in tumors

Cancer cells acquiring mutations in one or more pathways regulating survival and growth provide them with a selective growth advantage under conditions characterized by limited nutrients and oxygen. Accordingly, mutations in tumor cells include the EGFR, erythroblastic oncogene B2 (HER2/ERBB2), FGFR2, platelet-derived growth factor receptor (PDGFR), transforming growth factor beta receptor 2 (TGFβR2), MET oncogene, KIT proto-oncogene, Ras oncogene, Raf proto-oncogene, phosphatidylinositol 3-kinase cancer mutation (PI3KCA), and/or the phosphatase and tensin homolog deleted on chromosome ten (PTEN) genes [54]. These often mutated and constitutively expressed genes/pathways underscore the multi-step nature of cancer and the challenges ahead to achieve successful treatments for many tumor types. SCFAs delay tumor onset or block tumor progression by impacting upon these signaling pathways. Many cancer-associated mutations are difficult or impossible to correct, but the epigenetic modulation of these same pathways by SCFA intervention in the years prior to tumor development is expected to reduce the risk that such mutations will appear and be selected for over time.

### SCFAs and EGFR

EGFR is a transmembrane receptor that binds to epidermal growth factor (EGF) and transforming growth factor alpha (TGFα). Intrinsic tyrosine kinase activity transmits signaling downstream through MAPK, PI3K/Akt/mTOR, and Ras/Raf/MEK/ERK, resulting in cell proliferation, angiogenesis, and metastasis. SCFAs block EGFR signaling in colon cancer [109] and in breast cancer cell lines [110]. In HBx transgenic mice, SCFA feeding reduced the expression of EGFR by almost 8-fold in mice with dysplasia, and to a lesser extent in the livers of mice with HCC

(unpublished data). Given that EGF signaling is constitutively activated in many tumor types [111] including in the liver of patients with HCC [112, 113], EGF signaling may be an important step in liver cancer pathogenesis and that its attenuation by SCFAs may, in part, contribute to the delay in dysplasia and liver cancer [39]. Moreover, EGFR (ERBB1) is one of four molecules (ERBB1–4) that signal through JAK/STAT, Ras/ERK, c-Jun, and PI3K/Akt/mTOR [114]. Importantly, all these pathways are modulated by SCFAs (Fig. 3), suggesting that SCFAs could trigger apoptosis as well as block the development of multiple features of cancer, including extended survival, proliferation, angiogenesis, and metastasis.

Butyrate reduces the phosphorylation of Akt and up-regulates PTEN, which together attenuate PI3K/Akt signaling [115]. In this context, it is not surprising that PI3KCA, a constitutively active PI3K mutant, and loss of PTEN, are also found in many cancers, since they both contribute to carcinogenesis [116]. Diminished Akt signaling results in reduced mdm-2 activity. Since mdm-2 promotes ubiquitination and degradation of the tumor suppressor, p53, this should result in the stabilization of p53, cell cycle arrest, and DNA repair or apoptosis [116]. Diminished Akt signaling also results in reduced NF-κB activity and increased sensitivity of cells to apoptosis [116]. Thus, a cascade of pathways which are constitutively activated in tumors among various cell types are normalized by SCFAs so that homeostasis is re-established and the risks for tumor development and progression reduced.

### SCFAs and hepatocyte growth factor (HGF)/MET

Early tumors survive and grow in a hypoxic environment, and under such conditions, HIF-1 is expressed, which activates the transcription and expression of the MET proto-oncogene [117]. MET/HGFR is the receptor tyrosine kinase that binds to hepatocyte growth factor. Once engaged, MET signals through Ras, signal transducer and activator of transcription 3 (STAT3), β-catenin, and PI3K, resulting in sustained MAPK activation, promoting survival, proliferation, “stemness,” angiogenesis, and metastasis [118]. SCFAs epigenetically inhibit most of these pathways (Fig. 3) via HDAC inhibition, even though these same pathways are targets for driver mutations in carcinogenesis [54, 119]. HDAC inhibitors, including valproic acid and butyric acid, inhibit the production of HGF in fibroblasts induced by several ligands, including platelet-derived growth factor, EGF, and basic FGF. Given that HGF triggers c-MET signaling, inhibition of HGF production attenuated both MET signaling and the migration of HepG2 cells *in vitro*. This suggests that HDAC inhibition alters tumor–stromal interactions [120]. In addition, HGF/c-MET signaling promoted aerobic glycolysis (see below) through YAP/HIF1α signaling. Cross-talk of YAP/HIF1α with EGFR, ERBB2 (HER2), ERBB3 (HER3), and insulin-like growth factor 1-receptor (IGF-1R) signaling pathways, among others, amplifies the impact of HGF/c-MET activation [118]. Independently, butyrate has been shown to suppress the proliferation of eosinophilic precursor cells into eosinophilic leukemia cells. This is accomplished by inducing their differentiation into eosinophils and by down-regulating a fusion protein containing the PDGFR gene that expresses constitutive tyrosine kinase activity via HDAC inhibition [121]. Thus, SCFA attenuation of MET signaling impacts upon multiple downstream pathways that contribute to the malignant phenotype.

### SCFA modulation of other signaling pathways

Butyrate down-regulates the activity of ERK1/2 by blocking HDAC3 activity which then inhibits cell migration and metastasis [109]. Independent evidence from SCFA-treated HBx transgenic mice showed down-regulation of the Ras signaling molecules mitogen-activated protein kinase kinase (MEK1/2) and ERK1/2 that accompanied a significantly decreased frequency of liver cancer [39]. This is one of many examples that underscores the contribution of SCFA HDAC inhibitory activity to slowing cancer development.

The SCFA, acetate, triggers apoptosis in colon cancer cells by caspase 3 activation and DNA degradation, resulting in apoptosis [109]. Acetate also up-regulated expression of Fas and FasR on gastric adenocarcinoma cells which increased their sensitivity to cytotoxic T lymphocyte (CTL) killing and apoptosis [122]. In the colon cancer cell line, Colo320DM, SCFAs inhibited NF-κB signaling and decreased TNFα release from lipopolysaccharide (LPS)-treated neutrophils [123]. Since TNFα signals through NF-κB, this further attenuated NF-κB activity. Propionate may also trigger apoptosis in colorectal cancer cells by down-regulating the expression of arginine methyltransferase, although the mechanistic details remain to be elucidated [109]. In addition, propionate inhibited the growth of the pro-B murine tumor cell line Ba/F3, of the human histiocytic lymphoma U937, and of lymphoblast K562 cells through GPR43 signaling [124]. Propionate also triggered cell cycle arrest and apoptosis in the H1299 and H1703 lung cancer cell lines by reduction of survivin expression and elevated p21^WAF1/SDI1^ expression [125]. The expression of p21^WAF1^ was also increased by butyrate independent of p53 [126]. Since mutant p53 does not stimulate p21^WAF1^ expression, butyrate may partially compensate for mutant p53. These observations further highlight the potential utility of SCFAs as therapeutic agents against a cascade of signaling pathways that contribute to inflammation and possibly against cancer. In this context, SCFA therapeutics may be able to override the effects of selected oncogenic mutations through epigenetic regulation of the same pathways that are altered later by mutation during cancer pathogenesis.

## SCFAs and immunological homeostasis

In normal cells, ATP boosts the activation of mTOR signaling, which promotes the differentiation of naïve T cells into Th1, Th17, and CTLs, which are characteristically pro-inflammatory, but they also suppress the differentiation of bone marrow progenitors into antigen-presenting dendritic cells, which is anti-inflammatory [5]. SCFAs activate mTOR signaling in immune cells to provide anti-microbial and anti-tumor immunity, and they also promote the production of IgA mucosal immunity and systemic IgG production, while suppressing IgE-associated allergic responses [127, 128], which together also contribute to immunological homeostasis.

Impaired mucosal immunity often accompanies loss of gut integrity (e.g., due to decreased tight junction protein expression on colonocytes) and dysbiosis, which permits invasion of luminal microbes into Peyer’s patches and lamina propria, resulting in the appearance and progression of chronic inflammation, which may extend beyond the colon [129]. Loss of gut integrity (i.e., leaky gut) also results in the penetration of LPS from Gram-negative bacteria in the lumen through the gut wall. LPS binds to toll-like receptor 4 (TLR4), triggering innate immune responses via activation of NF-κB [130], the latter of which is strongly inhibited by SCFAs. Other potentially harmful compounds, such as elevated estrogen levels in the bloodstream, trigger chronic inflammatory responses that promote breast carcinogenesis and tumor progression [131, 132]. In the gut, bile acid metabolism is dependent upon the composition of the gut microbiome, with ursodeoxycholic acid promoting anti-inflammatory and anti-proliferative responses when reabsorbed by intestinal epithelia, while lithocholic and deoxycholic acids trigger elevated reactive oxygen and nitrogen species and activate NF-κB [133]. While the latter promotes colon carcinogenesis, SCFAs inhibit colonocyte proliferation and induce apoptosis by blocking mTOR/S6K1 signaling [134]. Butyrate also promotes the re-establishment of tight junctions and helps to re-establish the intestinal epithelial barrier by stimulating AMP-activated protein kinase (AMPK) [135], which is a metabolic sensor for increased intracellular AMP and ADP due to cellular stress by promoting protein catabolism to generate ATP. AMPK inhibits cell growth, promotes autophagy, suppresses anabolic pathways such as gluconeogenesis, contributes to the re-establishment of cell polarity, regulates the transcription of genes that alter cell metabolism, and reduces cell stress [136]. Thus, re-establishment of gut homeostasis promotes gut integrity and reduces or eliminates chronic inflammation.

### SCFAs, NF-κB, and chronic inflammation

**Fig. 4:**
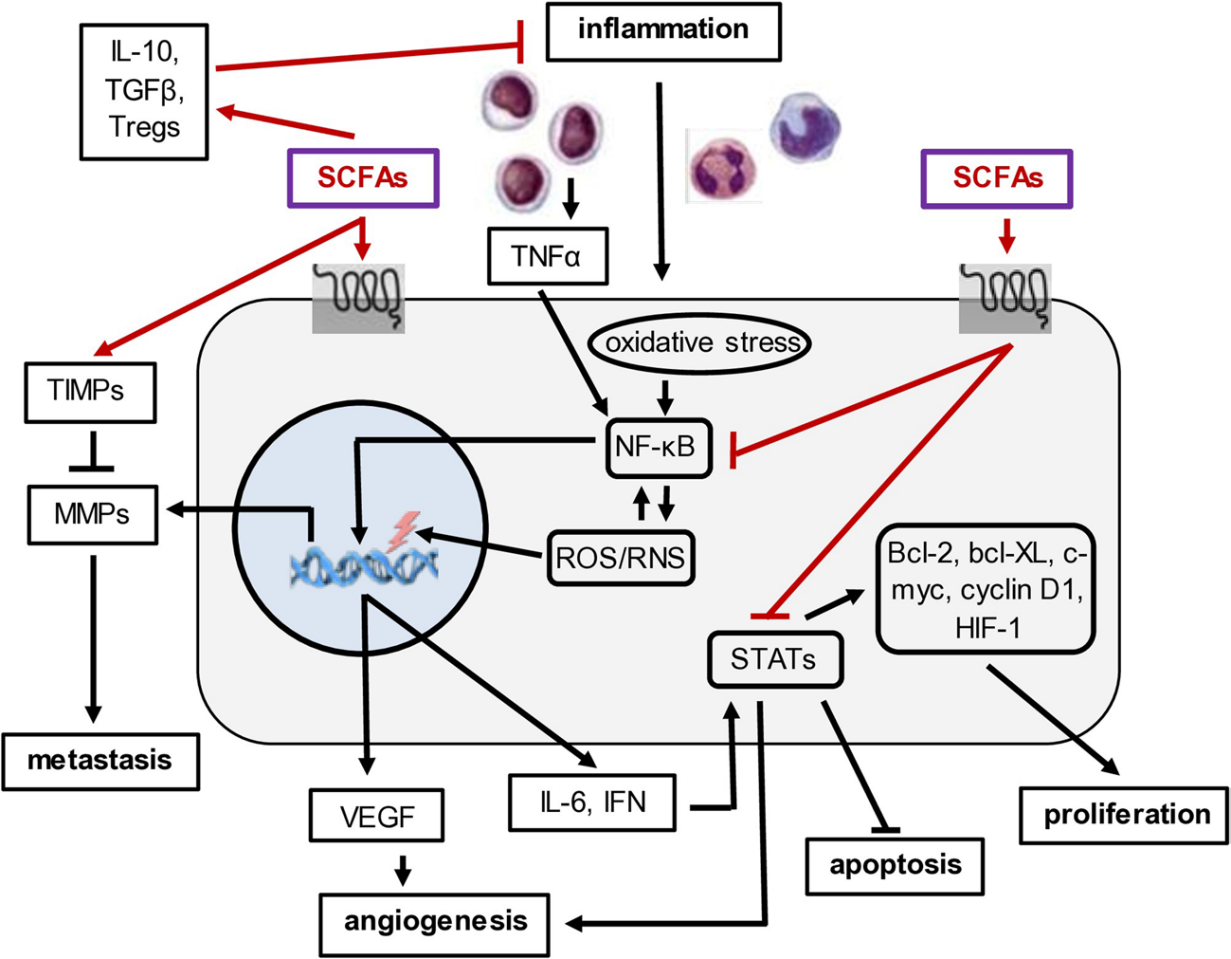
Role of SCFAs in attenuating NF-κB-associated inflammation and reducing the risk of cancer. An important component of chronic inflammation is the production of pro-inflammatory molecules such as TNFα, which is accompanied by persistent of oxidative stress and the production of oxygen and nitrogen free radicals. TNFα and oxidative stress stimulate the activity of NF-κB which then transcriptionally activates a large number of pro-inflammatory genes. The latter amplify free radical production which promotes the development of mutations in the host genome while further promoting NF-κB activity. In carcinogenesis, NF-κB up-regulates the expression of MMPs, which degrade extracellular matrix and facilitates metastasis. NF-κB up-regulates VEGF and angiogenesis and IFN signaling through intracellular STATs that transcriptionally alter gene expression to promote proliferation and block apoptosis. The role of SCFAs is that they promote the differentiation of naïve T cells into T regulatory cells and stimulate the production of anti-inflammatory cytokines. SCFAs also up-regulate TIMPS that attenuate metastasis and block both NF-κB and Stat signaling, thereby lowering the risk for progression to malignancy

There are a number of tumor types that arise on a background of chronic inflammation. For example, lung cancer can develop on a background of asbestosis, silicosis, or bronchitis. Bladder cancer can develop from cystitis, colorectal cancer from chronic bowel disease or Crohn’s disease, pancreatic cancer from pancreatitis, gastric cancer from gastritis, liver cancer from hepatitis, and ovarian cancer from pelvic inflammatory disease [137]. These and other inflammation-associated cancers are characterized by the presence and persistence of pro-inflammatory molecules in the affected tissue and tumor microenvironment, including cytokines, growth factors, and reactive oxygen and nitrogen species. This persistent oxidative stress results in the accumulation of mutations and genetic instability, thereby promoting cell proliferation, survival, angiogenesis, and metastasis. NF-κB activity is stimulated by oxidative stress, and this results in the up-regulated expression of anti-apoptotic proteins (e.g., bcl-2 and bcl-x_L_), promoters of DNA damage (e.g., reactive oxygen and nitrogen species), pro-inflammatory effectors (e.g., COX-2, TNF-α, IFNɤ, IL-6, Il-8, IL-17, IL-22, and IL-23), effectors of invasion and metastasis (e.g., matrix metalloproteinases), promoters of cell proliferation (e.g., c-myc and cyclin D1), and mediators of angiogenesis (e.g., vascular endothelial growth factor (VEGF) and angiopoietin) [127, 137, 138] (Fig. 4). IL-6, made from T cells, macrophages, and fibroblasts in the tumor microenvironment, stimulates signal transducer and activator of transcription 3 (STAT3) signaling that contributes to tumor progression [129]. Macrophage secretion of TNFα promotes inflammation and vascular permeability and constitutively activates oncogenic signaling pathways, such as Wnt and NF-κB [139]. Wnt activation by SCFAs results in differentiation while down-regulation of NF-κB largely blocks inflammation by reducing the expression of many NF-κB targets [72, 83]. Among them, pro-inflammatory cytokines are down-regulated by SCFAs and replaced by anti-inflammatory cytokines (e.g., IL-10 and TGFβ). At the cellular level, SCFAs promote the differentiation of naïve T cells to Tregs [15] (Fig. 4). SCFAs promote the expression of tissue inhibitors of matrix metalloprotinases (TIMPs), which attenuates cell migration and metastases [140]. Butyrate also inhibits STAT3 signaling, thereby down-regulating the expression of bcl-2, bcl-X_L_, c-myc, cyclin D1, and HIF-1, which results in decreased cellular proliferation and increased apoptosis in hypoxia [60]. Given that Ras signaling activates STAT3 and that SCFAs strongly inhibit Ras activity [39], this may also diminish STAT3 activation. STAT3 inhibition also blocks angiogenesis by down-regulating IL-8 and VEGF [60]. Further, butyrate blocks INFɤ stimulation of JAK2/STAT1 signaling [60], further underscoring its strong anti-inflammatory properties. The importance of targeting NF-κB derives from the fact that its constitutive activation is seen in many tumor types where it promotes cancer development and progression [141]. For example, application of SCFAs prior to the development of hepatitis B-associated HCC [39] and colitis-associated colorectal cancer [142] in preclinical models suggests that the immunomodulatory properties of SCFAs may reduce the risk of tumor development. Immunomodulation, however, involves a balance between pro- and anti-inflammatory immune responses. For example, SCFAs could promote T cell differentiation into either effector T cells that mediate pro-inflammatory responses to invading pathogens or anti-inflammatory Tregs that protect tissue integrity by extinguishing chronic inflammation often characteristic of tumor nodules and autoimmune diseases. In this context, epigenetic changes mediated by SCFAs in immune and other cell types are governed by the cellular and tissue environment (e.g., consisting of cytokines, nutrients, antigen composition and load, nuclear hormones, and other bacterial metabolites) over time [5, 68]. In addition, SCFA targets, like NF-κB, also has multiple functions. In the liver, for example, constitutive activation of NF-κB is both pro-inflammatory (promoting mutations) and hepatoprotective (promoting survival of virus infected cells), both of which contribute to carcinogenesis. Thus, the mechanisms whereby SCFAs act is context and target dependent, and this needs to be carefully considered in their development as therapeutic agents.

## SCFAs and the Warburg effect

Inflammation-associated oxidative stress also results in the activation of other transcription factors (besides NF-κB, STAT1, and STAT3) such as AP-1, HIFs, and nuclear factor erythroid 2-related factor 2 [137]. In a high-fiber diet, fiber is digested down to SCFAs, which up-regulate expression of HIFs to help preserve barrier function in the gut. Under normal conditions, an intact barrier provides a hypoxic environment in the intestinal lumen, which supports mostly obligate anerobic bacteria [143]. In this case, peroxisome proliferator-activated receptor γ (PPAR-γ)-dependent β-oxidation of SCFAs limits oxygen availability in the colon because butyrate, in the form of acetyl-CoA, provides the oxidative energy to maintain healthy colonocytes. In a low-fiber diet, oxygen is not reduced to water at the end of the electron transport chain, resulting in increased oxygen accumulation in the intestinal lumen, and the development of dysbiosis characterized by the outgrowth of facultative anerobes and the appearance of inflammation [144]. ATP production switches from oxidative phosphorylation to accelerated glycolysis (aerobic glycolysis or the Warburg effect) [10]. While this increases the risk of tumor development, restoration of SCFAs to physiological levels activates PPARγ signaling and attenuates the activation of pro-inflammatory NF-κB, AP-1, and STATs [145]. In this case, the higher levels of acetyl-CoA exhibit histone acetyltransferase (HAT) activity, thereby modulating chromosomal packing and the availability of chromatin for gene expression. In the nucleus, acetyl-CoA is also a substrate that is used to methylate the Notch target gene HEY1 in glioblastoma [146] as well as additional oncogenes in other tumor types [72, 147]. In tumor cells, butyrate is not used for energy production, but instead accumulates in the nucleus and alters patterns of host gene expression as an HDAC inhibitor, triggering cell cycle arrest or apoptosis [10]. Clonal expansion of immune cells in response to a particular antigenic stimulus also proceeds through aerobic glycolysis [148], and SCFAs as HDAC inhibitors also alter gene expression and immunomodulate, in part, by this mechanism. What is remarkable about SCFAs is their selective toxicity to cancer cells, while showing little or no toxicity to normal cells. As indicated above, this is based on differences in the way cancer and normal cells metabolize butyrate. This suggests that SCFAs could selectively target cancer cells while sparing surrounding cells in tissues and organs that have already experienced damage resulting from chronic inflammation.

## SCFAs and tumor suppressors

There is also evidence that SCFAs could compensate for loss of tumor suppressor function. For example, mutational loss of the tumor suppressor von Hippel–Lindau (VHL) protein results in elevated HIF-1 expression and stimulation of angiogenesis through the up-regulated secretion of VEGF, thereby promoting EMT [60]. However, these features are reversed by butyrate treatment [60], suggesting that epigenetic modulation of gene expression can sometimes overcome mutation inactivation of a tumor suppressor. Butyrate was also able to overcome the loss of the tumor suppressor, p53, by epigenetically up-regulating other negative growth regulators [149], such as p21^WAF^ [150]. Butyrate can also up-regulate the expression of silenced tumor suppressor genes [151] and restore cytoskeletal organization in APC mutated colon cancer cells [152]. In HBx transgenic mice that developed HCC, oral treatment with SCFAs significantly delayed the onset of liver cancer, in part, by up-regulating expression of the tumor suppressor, DAB2 [39]. SCFAs block Ras and Wnt signaling [39] by interfering with endocytic and vesicular trafficking [153]. DAB2 expression is suppressed by promoter hypermethylation in multiple tumor types [153] while SCFAs may reduce promoter methylation by suppressing the expression of several DNMTs [152]. Since genetic instability in the form of multiple mutations are characteristic of most tumor types, some of which may drive tumorigenesis, SCFA therapeutics may have utility in partially blocking cancer progression. Moreover, if some of the driver mutations occur prior to the appearance of frank malignancy, SCFA therapeutics may potentially delay or prevent tumor onset.

## SCFAs and immunotherapy

SCFAs promote the efficacy of anti-PD-1/anti-PD-L1 immunotherapy by several mechanisms. For example, tumor cells express the programmed death ligand 1 (PD-L1), which binds the PD-1 receptor on T cells, B cells, dendritic cells, and natural killer cells. This results in the suppression cell mediated anti-tumor immune responses [154]. PD-L1 expression is up-regulated by PI3K/Akt, STAT3, NF-κB, and HIF-1, all of which are suppressed by butyrate [120], suggesting that SCFAs are immuno-stimulatory, perhaps by permitting recovery from T cell exhaustion (see below). Butyrate also increases the immunogenicity of colon adenocarcinoma cells to CTL killing *in vitro* by promoting the expression of the major histocompatibility complex-1 (MHC-1) and intercellular adhesion molecule-1 [155]. In addition, the ability of SCFAs to improve gut barrier function and mediate the differentiation of naïve T cells into Tregs may suppress the immune-mediated toxicities often induced by immunotherapy, such as cytokine storm [155].

While immunotherapy with anti-PD-1/anti-PD-L1 has been fairly successful in treating liquid tumors (e.g., leukemia), it has been much less so with solid tumors because the microenvironment of solid tumors is immunosuppressive. It is also difficult for cell-based immunotherapy to penetrate solid tumors, especially those surrounded by a fibrotic capsule. In solid tumors, cancer cells expressing PD-L1 bind to T cells expressing PD-1, thereby down-modulating CTL effector function, resulting in T cell non-reactivity (exhaustion). In immunotherapy, addition of anti-PD-1 or anti-PD-L1 blocks the ability of tumor cells to trigger T cell exhaustion, thereby potentiating T cell mediated anti-tumor immunity. Tregs also express PD-1, which is up-regulated in the tumor microenvironment [156]. In liver cancer, for example, amphiregulin, an EGFR ligand produced by tumor cells stabilizes Treg cell function [157], which potentially facilitates tumor growth and metastases [158]. In the presence of anti-PD-1, Treg activity is further enhanced, resulting in the secretion of anti-inflammatory cytokines (e.g., IL-10 and TGFβ) [159] that inhibit CTL responses and promote therapy resistance, resulting in enhanced tumorgenicity. The relationship between the gut microbiota and immunotherapy was highlighted by findings that antibiotic treatment negatively impacted the clinical outcome of immunotherapy with anti-PD-1/PD-L1 [160] and that this was associated with a reduced abundance of gut bacteria that normally produce SCFAs [161, 162]. Subsequent work showed that SCFAs trigger T cell differentiation into T effector or Tregs depending upon the cytokine environment [155], so as to enhance CTL and chimeric antigen receptor T (CAR-T) cell activity in the tumor microenvironment and suppress a potential cytokine storm [155, 163]. Independent work showed that butyrate enhanced CTL activity by activating IL-12 expression via HDAC inhibition [164]. In both cases, the role of SCFAs would be to re-establish immunological homeostasis. In this way, the anti-tumor properties of SCFAs might possibly contribute as an adjuvant to cancer immunotherapy.

## Summary of SCFA role in targeting cancer hallmarks

The activities of SCFAs can be thought of as maintaining and/or re-establishing homeostasis by targeting genes and signaling pathways that contribute to multiple hallmarks of cancer. These hallmarks arise from a combination of epigenetic and genetic based changes in gene expression [55, 165–167]. Moreover, the link between epigenetic and genetic changes that define hallmarks of cancer is highlighted by observations indicating that genes encoding epigenetic regulatory proteins are often mutated in tumors [168]. Importantly, many of the pathways outlined above that are epigenetically modified by SCFAs are also considered hallmarks of cancer, but while the mutations which define some of these hallmarks are multiple and difficult to therapeutically correct, epigenetic modulation of these pathways by SCFAs to re-establish homeostasis, both in immune cells and in target tissues at risk for malignant transformation, may provide a window of opportunity that is not afforded by other approaches (Table 1). Although genetic instability and mutations are characteristic of most tumor types [52, 54], epigenetic changes in cancer have also been shown to contribute centrally to cancer pathogenesis [53, 56]. The reversible nature of epigenetic alterations in cancer pathogenesis may be an important key to more effective delay in cancer onset, as well as prevention and treatment [66].

**Table 1:** SCFAs and cancer hallmarks

Cancer hallmark	Examples of therapeutic approaches	Examples of associated mutations	SCFA targets that block hallmarks^*^
Sustained proliferative signaling	EGFR inhibitors	Overexpression or mutation in ERBB genes; Wnt and Ras mutations	⇩ EGFR signaling by ⇩ Ras, myc, PI3K/Akt, c-Jun, STAT3; ⇧ PTEN, p53, p300-β-catenin
Evading growth suppressors	CDK inhibitors	Rb, p53, and CDKi mutations	⇧ p21^WAF^, p57; p53, PTEN; ⇩ c-myc
Avoiding immune destruction	Immunotherapy	Absence of neoantigens	⇩ PI3K/Akt, Stat3, NF-κB and HIF-1 depress PD-L1
Enabling replicative immortality	Telomerase inhibition	TERT promoter mutations	⇧ Differentiation; ⇩ telomerase activity
Tumor-promoting inflammation	Anti-inflammatory drugs	Ras pathway mutations	⇩ NF-κB, STAT3, gut dysbiosis, Th_1_ cytokines; ⇧ Th_2_ cytokines
Activating invasion and metastases	HGF/c-MET inhibitors	Mutations in the MET oncogene	⇧ Wnt, TIMPs, differentiation; ⇩ STAT3, MMPs, NF-κB, Ras, Hippo
Inducing angiogenesis	VEGF signaling inhibitors	Notch mutations	⇩ VEGF, Akt, Rho, STAT3, Ras, Hippo
Genome instability and mutations	PARP inhibitors	Mutations in DNA repair genes	⇩ Gut dysbiosis and ROS/RNS
Resisting cell death	Proapoptotic compounds	Wnt and Ras mutations	⇧ bax, FasL, p300-β-catenin; ⇩ bcl-2
Deregulating cellular energetics	Aerobic glycolysis inhibitors	Mutations in glycolytic enzyme encoding genes	⇧ PPARγ, HDACi, and HAT activity

^*^Up and down arrows mean that SCFAs up- or down-regulate expression of the gene(s) encoding the protein(s) directly to the right of the arrow

The ability of SCFAs to inhibit the hallmark of sustained proliferation [54, 60, 69, 77] and promote differentiation of cells undergoing malignant transformation may delay or prevent the development of cancer (Table 1). The inhibition of sustained proliferation with cell cycle arrest or apoptosis [60, 109, 122, 125] blocks the hallmark of resistance to cell death. In cancer cells, the hallmark of deregulated cellular energetics (aerobic glycolysis) is altered by butyrate. Butyrate enters the nucleus as an HDAC inhibitor and triggers the expression of genes that arrest cell growth and mediate apoptosis [10]. This will also deprive incipient cancer cells from surviving in a hypoxic environment long before tumors are large enough to be clinically detectable. Reduction of inflammation by SCFAs prior to and after tumors appearance, thereby reducing the hallmark of chronic and tumor-associated inflammation [3, 45, 60, 72, 83], will reduce the levels and persistence of free radicals that contribute to the appearance of these mutations. This will mitigate the hallmark of genome instability that favors the selection of driver genes over time. Epigenetic modulation that reduces the development of hypoxia in early neoplasia also blocks the hallmark of tumor associated angiogenesis [60]. SCFAs also up-regulate the expression of several tumor suppressor proteins [39, 72, 116, 169]. In this case, malignant cells are no longer able to circumvent the hallmark of evading growth suppression, which then results in increased growth arrest and apoptosis. In prostate, uterine, cervical, and liver cancer cells, butyrate inhibited telomerase activity [170–173], thereby depriving cells of the hallmark enabling replicative immortality. In the context of immunotherapy, SCFAs may act to overcome T cell exhaustion and reactivate CTL activity [154, 164] and enhance the cytotoxic activity of CAR-T cells through stimulation of mTOR (metabolic reprogramming) and via HDAC inhibitory activity (epigenetic reprogramming). This has been shown in pancreatic and melanoma cells, in which SCFA treatment overcomes the hallmark of tumor cells avoiding immune destruction [174]. However, given the genetic and phenotypic heterogeneity of most cancers, immunotherapy may have limited utility in that it will select for resistance [175]. As a consequence, recent work has focused on the development of combination therapies [176] and it is possible that SCFAs can be part of that combination. Cell migration and metastasis, another hallmark of cancer, is also inhibited by the HDACi activity of butyrate [109]. Thus, SCFAs epigenetically block hallmarks of cancer that were previously defined by driver gene mutations that resulted in the same changes in cellular phenotype. The broad activity of SCFAs suggests that they are especially suited for reducing the risk of cancer development and progression under many circumstances, although this remains to be evaluated in human clinical trials.

## SCFAs and miRNAs in cancer pathogenesis

Host epigenetics, especially miRNAs, participate in physiological functions related to maintaining intestinal homeostasis by regulating gut microbiota. For example, the miR-21-5p expression in intestinal epithelial cells regulates intestinal epithelial permeability through ADP ribosylation factor 4 [177]. In this context, SCFAs facilitate the re-establishment and maintenance of gut integrity, thereby restoring normal gut bacteria. Host-derived miRNAs impact intestinal homeostasis by regulating the growth and structure of microbial communities. This provides a new perspective for maintaining intestinal health [178], that is, in part, regulated by SCFAs.

Altered expression of miRNAs also contributes to the pathogenesis of many tumor types [178]. Part of the epigenetic properties of SCFAs involves the altered expression of selected miRNAs [60, 178–180]. For example, transfection of miR-16, miR-34a, and miR-449a into HeLa cells trigger senescence and apoptosis, suggesting they act as tumor suppressors [179]. Butyrate alters the expression of numerous miRNAs that impact upon oncogenesis-related signaling pathways. Butyrate blocks expression of miR-106B, resulting in the up-regulation of p21^WAF1^, which triggers cell cycle arrest [180]. Butyrate also stimulates the expression of miR-22 and miR-203, both of which inhibit cyclin-dependent kinases and cell proliferation, thereby also contributing to cell cycle arrest [60]. In another study, butyrate changed the expression of 44 miRs in the colon cancer cell line HCT-116 [180]. Independent observations showed that 33 miRs were altered by butyrate in the non-small-cell lung cancer cell line A549, resulting in decreased cell proliferation and migration [181]. Given that each miRNA will impact the expression of multiple host genes, it is not surprising that the altered expression of multiple miRNAs by SCFAs will have a major impact upon the pathogenesis of multi-step carcinogenesis.

## Future perspectives

SCFAs epigenetically target multiple signaling pathways containing molecules that are often mutated in cancer. These mutation-carrying genes and pathways are often drivers of carcinogenesis [52] and mediate many of the hallmarks of cancer [55]. The fact that SCFAs epigenetically target many of these same driver genes and pathways underscores their potential relevance as therapeutic agents. Unlike other approaches for treating cancer, characterized by drugs that often target a single pathway or molecule, SCFAs simultaneously target multiple pathways reflecting multi-step carcinogenesis, which suggests that they will have a sustained anti-tumor effect. In addition, it is likely that the anti-inflammatory properties of SCFAs will have their greatest impact prior to tumor development among inflammation-associated cancers, since reducing inflammation reduces cancer risk, suggesting a “prevention by delay” approach would be feasible as a means of cancer control [36]. If so, at-risk patients could be treated prior to cancer appearance, significantly reducing morbidity and mortality. There is already an indication that a sustained intake of a high-fiber diet, where fiber is digested to SCFAs by gut bacteria, could be a viable approach of achieving cancer control for at least some tumors [75, 182]. Chronic inflammation is damaging to organs, which makes the application of immunotherapy challenging, while SCFAs are most likely to be efficacious at doses and for durations which do not trigger toxicity to already compromised tissues and organs. While Tregs are induced by SCFAs and promote tumorigenesis, this is countered by properties of SCFAs that promote cellular differentiation (of would be tumor cells) and immunological homeostasis (which would extinguish inflammation). In this context, dysbiosis (via rDNA sequencing) and decreased SCFA levels in feces may have prognostic value years prior to the onset of cancer. Monitoring of fecal SCFA levels may also be of value in following chronic inflammatory diseases treated with monoclonal antibodies, immunotherapy, or by other approaches. Combination therapies will need to be considered when applying SCFAs for the treatment of solid tumors in human clinical trials and beyond. One of the limitations of SCFAs in clinical trials is their short half-life in blood, which is on the order of minutes for butyrate and propionate. A possible approach to solve this is the encapsulation of SCFAs into nanoparticles that can be used in combination with theranostics, the latter of which can be used to image tumor-bearing patients [183, 184]. Nanoparticles are currently being developed to limit systemic toxicity of cancer therapeutic compounds [184, 185], but SCFAs are generally regarded as safe and nontoxic at therapeutic doses. Although SCFAs promote the differentiation of naïve T cells to Tregs, which would attenuate anti-tumor immune responses, solid tumors often trigger T cell exhaustion, allowing them to escape immune elimination. Fortunately, SCFAs are taken up and epigenetically trigger differentiation or apoptosis in tumor but not normal cells, suggesting they provide an alternative means of anti-tumor therapeutics. Systemic distribution of SCFAs also have the potential to treat metastatic nodules without the limitations posed by nanoparticles (e.g., non-targeted distribution causing low signal-to-noise ratio for diagnostics, complex fabrication, reduced-biocompatibility, decreased photostability, and systemic toxicity) [184, 185], although the latter can still be very useful for imaging in the context of tumor diagnostics and monitoring response to treatment. Thus, SCFAs can be combined to complement other therapies to lower the risk of cancer development and to treat tumor bearing patients. Given the multi-step nature of cancer pathogenesis, system biology will aid in the design of combination therapies to delay the onset and progression of cancer [186]. The properties of SCFAs, as outlined herein, are likely to contribute importantly to this approach.

## Abbreviations

AhR: Aryl hydrocarbon receptor
AML: Acute myeloid leukemia
AMPK: Adenosine monophosphate-activated protein kinase
AP-1: Activator protein-1
APC: Adenomatous polyposis coli
bcl-2: B cell lymphoma 2
CBP: cAMP response element binding protein
CAR-T cells: Chimeric antigen receptor T cells
CDKi: Cyclin-dependent kinase inhibitor
CLD: Chronic liver disease
COX-2: Cyclooxygenase-2
CSC: Cancer stem cells
CTL: Cytotoxic T lymphocyte
DAB2: Disabled 2
DNMT: DNA methyltransferase
ECM: Extracellular matrix
EGF: Epidermal growth factor
EGFR: Epidermal growth factor receptor
EMT: Epithelial to mesenchymal transition
EPCAM: Epithelial cell adhesion molecule
ERBB2: Erythroblastic oncogene B2
ERK1/2: Extracellular signal–regulated kinases 1/2
FFAR: Free fatty acid receptor
FGF: Fibroblast growth factor
FGFR2: Fibroblast growth factor receptor 2
Gli: Glioma-associated oncogene
GPCRs: G-protein coupled receptors
HAT: Histone acetyltransferases
HBx: Hepatitis B x antigen
HBV: Hepatitis B virus
HCA2: Hydroxycarboxylic acid receptor 2
HCC: Hepatocellular carcinoma
HDACi: Histone deacetylase inhibitor
Hh: Hedgehog
HIF-1a: Hypoxia-inducible factor-1 alpha
HSC: Hematopoietic stem cells
IEL: Intraepithelial lymphocytes
IFNɤ: Interferon gamma
IgA: Immunoglobulin A
IgE: Immunoglobulin E
IGF-1: Insulin-like growth factor 1
IGF-1R: Insulin-like growth factor-1 receptor
IgG: Immunoglobulin G
IL-6: Interleukin-6
JAK/STAT: Janus kinase/signal transducer and activator of transcription
LPS: Lipopolysaccharide
MAPK: Mitogen-activated protein kinase
miRNA: MicroRNA
MMPs: Matrix metalloproteinases
mTOR: Mammalian target of rapamycin
MCT-1: Monocarboxylate transporter 1
mdm-2: Mouse double minute 2
MEK: Mitogen-activated protein kinase kinase
MHC-1: Major histocompatibility complex-1
NF-κB: Nuclear factor kappa B
NFAT: Nuclear factor of activated T cells
OAT: Organic anion transporter
PD-1: Programmed cell death protein-1
PD-L1: Programmed cell death protein-1 ligand
PDGFR: Platelet-derived growth factor receptor
PI3K: Phosphoinositide 3-kinase
PPAR: Peroxisome proliferator-activated receptor
PTEN: Phosphatase and tensin homolog deleted on chromosome ten
rDNA: Ribosomal DNA
RNS: Reactive nitrogen species
ROS: Reactive oxygen species
SCFAs: Short-chain fatty acids
SMCT-1: Sodium-coupled monocarboxylate transporter-1
TCF/LEF: T-cell factor/lymphoid enhancer factor
TGFα: Transforming growth factor alpha
TGFβ: Transforming growth factor beta
TGFβR2: Transforming growth factor beta receptor 2
TIMPs: Tissue inhibitor of matrix metalloproteinases
TLR4: Toll-like receptor 4
TNFα: Tumor necrosis factor alpha
Tregs: T regulatory cells
VEGF: Vascular endothelial growth factor
VHL: von Hippel–Lindau
YAP: Yes-associated protein
